# Fluoxetine Exerts Age-Dependent Effects on Behavior and Amygdala Neuroplasticity in the Rat

**DOI:** 10.1371/journal.pone.0016646

**Published:** 2011-01-31

**Authors:** Judith R. Homberg, Jocelien D. A. Olivier, Tom Blom, Tim Arentsen, Chantal van Brunschot, Pieter Schipper, Gerdien Korte-Bouws, Gilles van Luijtelaar, Liesbeth Reneman

**Affiliations:** 1 Department of Cognitive Neuroscience, Centre for Neuroscience, Donders Institute for Brain, Cognition, and Behaviour, Radboud University Nijmegen Medical Centre, Nijmegen, The Netherlands; 2 Department of Radiology, Academic Medical Centre, University of Amsterdam, Amsterdam, The Netherlands; 3 Department of Biological Psychology, Centre for Cognition, Donders Institute for Brain, Cognition, and Behaviour, Radboud University Nijmegen, Nijmegen, The Netherlands; 4 Division of Pharmacology, Utrecht Institute for Pharmaceutical Sciences, Rudolf Magnus Institute of Neuroscience, Utrecht University, Utrecht, The Netherlands; Institut National de la Santé et de la Recherche Médicale, France

## Abstract

The selective serotonin reuptake inhibitor (SSRI) Prozac® (fluoxetine) is the only registered antidepressant to treat depression in children and adolescents. Yet, while the safety of SSRIs has been well established in adults, serotonin exerts neurotrophic actions in the developing brain and thereby may have harmful effects in adolescents. Here we treated adolescent and adult rats chronically with fluoxetine (12 mg/kg) at postnatal day (PND) 25 to 46 and from PND 67 to 88, respectively, and tested the animals 7–14 days after the last injection when (nor)fluoxetine in blood plasma had been washed out, as determined by HPLC. Plasma (nor)fluoxetine levels were also measured 5 hrs after the last fluoxetine injection, and matched clinical levels. Adolescent rats displayed increased behavioral despair in the forced swim test, which was not seen in adult fluoxetine treated rats. In addition, beneficial effects of fluoxetine on wakefulness as measured by electroencephalography in adults was not seen in adolescent rats, and age-dependent effects on the acoustic startle response and prepulse inhibition were observed. On the other hand, adolescent rats showed resilience to the anorexic effects of fluoxetine. Exploratory behavior in the open field test was not affected by fluoxetine treatment, but anxiety levels in the elevated plus maze test were increased in both adolescent and adult fluoxetine treated rats. Finally, in the amygdala, but not the dorsal raphe nucleus and medial prefrontal cortex, the number of PSA-NCAM (marker for synaptic remodeling) immunoreactive neurons was increased in adolescent rats, and decreased in adult rats, as a consequence of chronic fluoxetine treatment. No fluoxetine-induced changes in 5-HT_1A_ receptor immunoreactivity were observed. In conclusion, we show that fluoxetine exerts both harmful and beneficial age-dependent effects on depressive behavior, body weight and wakefulness, which may relate, in part, to differential fluoxetine-induced neuroplasticity in the amygdala.

## Introduction

Selective serotonin reuptake inhibitors (SSRIs) are among the most widely prescribed drugs in psychiatry. While numerous trials have shown robust safety of SSRIs in adults, limited data are available on their short- and long-term safety in adolescents. Yet, the number of children for whom anti-depressants are prescribed has increased during the last decade [1]. Fluoxetine is the only SSRI registered for treatment of depression in the paediatric population. Some alarming studies have reported that children and adolescents may experience increases in suicidal ideation and behavior, as well as agitation, depression and anxiety [2]–[6]. A meta-analysis revealed that the younger the children were the greater the risk was of suicidal thoughts or attempts [7]. Based on some of these reports, the Federal Drug Agency and European Medicines Agency stated in 2004 that SSRIs were *contraindicated* for treating depression in children and adolescents. However, in 2006 fluoxetine was approved in children aged 8 years and older for treatment of moderate to severe depression [8].

Some recent rodent studies have elaborated the human findings. Mason et al. [9] showed that subchronic fluoxetine treatment (10–20 mg/kg) during the 5^th^ week of age had no effect on depression-like behavior in mice. Also chronic adolescent fluoxetine treatment (10–18 mg/kg) between 3 and 7 weeks of age did not affect adult measures of anxiety-, fear- or stress-related behaviors in mice [10]. However, another mouse study reported that adolescent fluoxetine treatment (7.5–16 mg/kg) at 4–9 weeks of age prevented increased depression-related immobility in the forced swim test following maternal separation stress [11]. Further, Oh and colleagues [12] showed that juvenile mice treated with fluoxetine (2–4 mg/kg) displayed paradoxical anxiogenic responses, but these effects disappeared upon drug discontinuation. Using rats, it was shown that adolescent fluoxetine (10 mg/kg) exposure resulted in impaired visual discrimination, after a wash-out period of 14 days [13]. Finally, Iñiguez et al. [14] reported that exposure to fluoxetine (10 mg/kg) from postnatal day (PND) 35 to 49 was associated with decreased responsiveness to forced swimming stress, increased sensitivity to natural reward and anxiety-eliciting situations, as well as deficits in sexual behavior, during adulthood. Adult fluoxetine exposure alleviated the increased anxiety induced by the adolescent fluoxetine [14], suggesting that adolescent and adult fluoxetine exposure can have opposing effects. Collectively, the literature on adolescent SSRI exposure in rodents is rather mixed, with negative (no drug effect), beneficial (decreased stress responsiveness) and adverse (increased sensitivity to anxiety-eliciting conditions) outcomes.

The present study aimed to increase the understanding of the age-related outcomes of adolescent fluoxetine exposure. Since the mixed results reviewed above may be due to differential wash-out periods and ages of testing, and the adverse effects of adolescent fluoxetine exposure in humans are manifested particularly short after the start of treatment [2]; [15], we specifically focussed on the time period shortly after the wash-out of fluoxetine, when neuroplastic changes may have established. We also aimed to establish whether (nor)fluoxetine levels in rats were in the clinical range, and to extend the behavioral repertoire sensitive to SSRI treatment. Therefore, we not only included emotion-related tests, but also tests for sensorimotor integration and sleep/wake patterns. Finally, our goal was to shed light on potential neuroplastic changes underlying the age-dependent effects of SSRIs. To these ends we treated adolescent rats from PND 25 to 49, and adult rats from PND 67 to 88, orally with 12 mg/kg fluoxetine, and tested the animals 7–14 days later, when fluoxetine had been washed out. The adolescent window we used approximates mid-childhood through adolescence in humans [16]–[18]. The animals were tested in a series of tests measuring emotional behavior, namely the open field test (novelty-induced locomotor activity), elevated plus maze (EPM) test (anxiety), forced swim test (behavioral despair), and the acoustic startle response. In addition, sensorimotor integration (prepulse inhibition; PPI) and sleep/wake behavior (electroencephalography) were measured. We observed both harmful (behavioral despair; no effect on wakefulness) and beneficial (no anorexic effect) outcomes of fluoxetine treatment during adolescence compared to adulthood. Age-dependent effects on the acoustic startle response and prepulse inhibition were also observed, but not in the elevated plus maze test. Exploratory behavior was not affected by fluoxetine.

There is accumulating evidence that SSRIs exert their effects through neuroplastic changes (for review see [19]–[21]). Because the adolescent brain is more plastic than the adult brain, differential neuroplastic effects in adolescent and adult rats could underlie age-dependent effects of fluoxetine. To elucidate some of the mechanisms underlying our behavioral observations, we assessed plasma levels of fluoxetine and the metabolite norfluoxetine. In addition, we conducted a series of immunohistochemical stainings focussing on the 5-HT_1A_ receptor and PSA-NCAM in the dorsal raphe nucleus (origin of serotonergic cell bodies), amygdala, and medial prefrontal cortex (mPFC), brain areas playing a central role in emotional, cognitive and sensory information processing and responsive to the (therapeutic) effects of antidepressants [22]–[26]. The 5-HT_1A_ receptor is strongly implicated in the actions of SSRIs [27]; [28], and plays a role in neuroplasticity as well [29]. PSA-NCAM is the polysialylated (PSA) form of the neural cell adhesion molecule (NCAM) that is involved in neurite and synaptic remodeling. It is modified by chronic fluoxetine exposure in a region-dependent manner [25]. We observed that amygdalar PSA-NCAM, but not 5-HT_1A_ receptor, immunoreactivity was differentially affected by fluoxetine in adolescent and adult rats.

## Materials and Methods

### Animals

All experiments were approved by the Committee for Animal Experiments of the Radboud University Nijmegen Medical Centre, Nijmegen, The Netherlands, and all efforts were made to minimize animal suffering and to reduce the number of animals used.

Wistar Unilever (WU) male rats (Harlan, Horst, The Netherlands) were 21 and 63 days of age at arrival. Since they were obtained from the same experimental animal supplier and both age groups arrived at the animal facility 4 days before the start of the treatment, the background and history of both age groups was similar. The animals were housed two per cage (Macrolon® 40×25×15 cm) in temperature controlled rooms (20±2°C). After 4 days of acclimatisation, the rats were daily treated with 12 mg/kg fluoxetine (Pharmacy Radboud University Nijmegen Medical Centre) or 1% methylcellulose (Genfarma B.V. Maarssen, the constituent of the fluoxetine pills that were used) by oral gavage for 21 days. Rats treated during PND 25–49 are referred to as the adolescent group, and rats treated during PND 67–88 represent the adult group. Body weight was monitored daily throughout the treatment. From 7 days after the last injection the animals were tested as described below. Separate groups of animals were used for 1) the open field and elevated plus maze tests (10 adolescent methylcellulose, 10 adolescent fluoxetine, 10 adult methylcellulose, and 10 adult fluoxetine treated rats), 2) the acoustic startle/prepulse inhibition and forced swim tests (10 adolescent methylcellulose, 10 adolescent fluoxetine, 10 adult methylcellulose, and 10 adult fluoxetine treated rats), and immunohistochemistry (4 adolescent methylcellulose, 4 adolescent fluoxetine, 5 adult methylcellulose, and 5 adult fluoxetine treated rats), and 3) sleep-wake behavior and plasma (nor)fluoxetine levels (7 adolescent methylcellulose, 7 adolescent fluoxetine, 7 adult methylcellulose, and 7 adult fluoxetine treated rats). Consecutive tests were separated by 2 days (see table 1 for time schedule). Housing and testing (between 09.00 a.m. and 16.00 p.m.) took place under a standard 12-hr day/night cycle (lights on at 07.00 a.m.), except for animals in experimental group 3 (sleep/wake behavior), which were housed directly after arrival in the animal facility under a reversed day/night cycle (lights off at 07.00 a.m.) and were tested over 24 hrs.

**Table 1:** Time schedule of experiments.

	5 hrs after last injection	7 days after last injection	10 days after last injection	14–17 days after last injection
**Group 1**		Open field	Elevated plus maze	
**Group 2**		Prepulse inhibition	Forced swim test	Immunohistochemistry
**Group 3**	Blood collection	Blood collection		EEG/EMG

Each group consisted of adolescent and adult rats treated with either fluoxetine or methylcellulose.

### Surgery

Two weeks before testing, rats were implanted, under complete anesthesia (isoflurane), with a standard cortical tripolar electroencephalography (EEG) electrode set (Plastics One MS-333/2-A, Plastic Products, Roanoke, VI, USA) and a bipolar electromyography (EMG) electrode set (Plastics One MS 303/71). EEG electrodes were placed in the frontal cortex and in the parietal region, with coordinates A 2.0, L 3.5 and A −6.0, L 4.0, respectively (with skull surface flat and bregma zero-zero; [30]), while the third earth electrode was placed in the cerebellum. The EMG electrode was subcutaneously placed in the dorsal neck muscles. After surgery the rats were individually housed and allowed to recover for two weeks.

### Behavior

#### Novelty-induced locomotor activity

Novelty-induced locomotor activity was recorded by video tracking in Phenotyper® cages (Noldus Information Technology, Wageningen, The Netherlands). The cages (45×45×45 cm), made of transparent Perspex walls and a black floor, were equipped with a feeding station and two drinking bottles. Each cage had a top unit containing a built-in digital infrared-sensitive video camera, infrared lighting sources, and hardware needed for video tracking. The rats were placed in the Phenotyper cages [31] and total distance moved (cm) was monitored for 1 hr.

#### EPM

The apparatus, made of polyvinylchloride, was elevated to a height of 50 cm with two open (50×10; 2.5 lux) and two enclosed (50×10×40; 0.2 lux) arms. As described earlier [32], rats were allowed to freely explore the maze for 5 min. Behavior was registered automatically by a computerized system (Plus Maze^©^, Nijmegen, The Netherlands). Results were expressed as the mean of time spent (s) in open arms.

#### Forced swim test

Cylindrical glass tanks (50 cm tall×18 cm diameter), filled to a depth of 30 cm with 22 (+/−1)°C water, were used. After a 15-min water experience on day 1, the animals were tested 24 hrs later in the water cylinders for 5 min [32]. The movements of the rats were videotaped for off-line measurement. ‘Immobility’ was defined as making no movements for at least 2 seconds or making only those movements that were necessary to keep the nose above the water.

#### Startle response and PPI

The acoustic startle chambers consisted of a Plexiglas tube (8.2 cm in diameter, 25 cm in length) with a piezoelectric accelerometer mounted beneath the tube. The acoustic stimuli were delivered by the PSR2 computer software, via a speaker that was placed 10 cm above the tube. The software converted the accelerometer measurements into a digital signal. The background noise was 70 dB. Each session started with 5 min acclimatisation, followed by ten blocks of 5 trials consisting of one 120 dB startle stimulus (basal amplitude), a no stimulus condition, and pre-pulse startle stimuli of 3, 5 or 10 dB above background (delivered pseudo-randomly). The prepulses were always followed by the 120 dB stimulus after 100 ms. All stimuli were delivered for 20 ms. The interval between each trial was 10/20 s. The startle amplitude was calculated as an average of the 10 trials of the startle trial and the three different prepulse trials. The % PPI was calculated as follows: 100 - (startle amplitude/basal startle amplitude) ×100.

#### Analysis of wake/sleep patterns

Rats were kept in a Perspex recording cage (30×25×35 cm) equipped with a passive infrared movement detector (Lunar PR 360° ceiling mount PIR, Rokonet Industries, U.S.A.) attached to the ceiling of the cage. Rats, chronically provided with cortical EEG electrodes and nuchal EMG electrodes, were connected to a swivel, which allowed free movement in their recording cage. EEG and EMG signals were amplified, band-pass filtered (EEG 1–100 Hz; EMG 1–1000 Hz), a notch filter eliminated 50 Hz, sampled at 256 Hz and stored on disk with the aid of a WINDAQ data acquisition system (DATAQ Instruments, Akron, OH, USA). We analyzed 4 hrs (17.30–18.30, 19.30–20.30, 5.30–6.30 and 7.30–8.30 hrs) from each rat, using the WinDaq Waveform Browser (DATAQ q Instruments, Akron, OH, UA). Sleep and wake states were visually scored according to conventional criteria based on EEG and EMG [33], supplemented by the PIR scores. Wakefulness was characterized by a small amplitude, fast frequency EEG together with a high amplitude and/or a rapidly changing EMG and or PIR; non-REM sleep by a large amplitude, low frequency EEG together with a moderate and relatively constant EMG and low PIR; sleep spindles by a pattern of symmetrical rhythmic waves, a waxing and waning morphology with round peaks and valleys and a dominant frequency of 11–15 Hz, minimal duration 0.5 sec [33]; whereas REM sleep was characterized by a low voltage, high frequency EEG with predominant beta-theta activity, and a low amplitude EMG (atonia with occasional twitches) and a low PIR score. Reported is the time (minutes) spent in each state.

### Measurement of Fluoxetine and Norfluoxetine in blood plasma

#### Plasma collection

5 hrs and 1 wk after the last fluoxetine injection blood samples were collected in Microvette CB 300 (Sarstedt, Germany) tubes through a tail cut. Blood plasma was obtained by centrifugation of the blood at 4°C at 4000 rpm for 15 min. Supernatant was stored at −20°C until use.

#### HPLC

50–100 µl of the plasma samples, containing fluvoxamine as internal standard, were extracted as described by Duverneuil et al. [34]. The concentration of fluoxetine and norfluoxetine was determined by HPLC, which has been described previously [35]. The mobile phase consisted of a buffer containing 20 mM citric acid and 20 mM phosphoric acid (pH adjusted to 3.8 with NaOH) mixed with acetonitril (55∶45). Separation was performed at 32°C using a flow rate of 0.8 ml/min. The concentration of each compound was calculated by comparison with both the internal and external standards. The limit of detection (signal/noise ratio 3∶1) was 15 ng/ml in 100 µl plasma samples.

#### Reagents

Norfluoxetine and fluoxetine were purchased from Sigma, HPLC grade acetonitril and hexane from Biosolve B.V. (Valkenswaard, The Netherlands), isoamylalcohol and hydrochloric acid from Merck (Darmstadt, Germany), and sodiumhydroxide, phosphoric acid and citric acid monohydrate were obtained from Acros (Geel, Belgium).

### Immunohistochemistry

7 Days following the forced swim test, rats of group 2 were deeply anesthetized and perfused transcardially with 0.1 M PBS, pH 7.3, followed by 400 ml 4% paraformaldehyde dissolved in 0.1 M PB, pH 7.2. Immunostaining was performed as previously described [32], using 5-HT_1A_ (1∶250; Santa Cruz Biotechnology, Santa Cruz, CA, USA), or PSA-NCAM (1∶8.000; Millipore, Billerica, MA, USA) antisera.

#### Quantification

Numbers of PSA-NCAM immunopositive cells were quantified [26] using the software program Neurolucida (MicroBrightfield Inc, Williston, VT, USA), and 5-HT_1A_ immunoreactivity was quantified using Image J, a public domain image processing program (http://rsb.info.nih.gov/ij/) that assessed the intensity of immunostaining [25]. Intensity was corrected for background intensity, and expressed as relative optical density (O.D.). Target areas included the dorsal raphe nucleus (bregma −7.68 [30]), the basolateral amygdala (bregma −2.6 to −3.3; [30]) and mPFC (prelimbic cortex; bregma +4.68 to +3.0; [30]). The latter two subregions were based on reported fluoxetine effects on PSA-NCAM expression [25]. Immunoreactivity was assessed in homologous square fields (using a grid overlay with a size of 100×100 µm) that displayed a representative density of stained cells, at 20x magnification.

### Statistical analysis

All statistical analyses were performed using SPSS version 16.0 (SPSS Inc., Chicago, IL, USA). Data were analyzed using one-way ANOVA followed by Bonferroni *post-hoc* testing (time effect sleep/wake behavior), two-way ANOVA (novelty-induced locomotor activity, anxiety, behavioral despair, acoustic startle, PPI, immunohistochemistry) or repeated measures ANOVA (sleep/wake behavior, body weight). Interaction effects were further analysed using two-way ANOVA (sleep/wake patterns) and Student t-tests. Probability values of p<0.05 were considered significant. NS  =  not significant.

## Results

### Bodyweight

**Figure 1:**
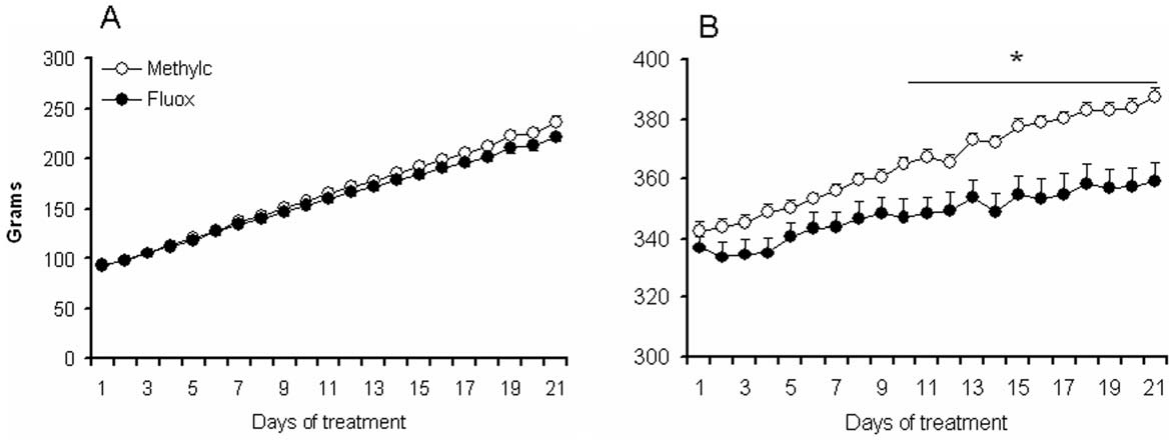
Effect of fluoxetine on body weight in adolescent and adult rats. Data are presented as mean ± S.E.M. body weight (g) in adolescent rats (A) and adult (B) rats (n = 10). 21 Days of fluoxetine treatment had no effect on bodyweight of adolescent rats, but reduced bodyweight in adult rats. ^*^p<0.05 fluoxetine *versus* methylcellulose in age group.

The bodyweight of fluoxetine and methylcellulose treated animals (group 1) across the fluoxetine/methylcellulose treatment is shown in figure 1A (adolescents) and 1B (adults). Starting weight was not different in the adolescent (t_(1,18)_ = 0.6, NS) and adult (t_(1,18)_ = 0.474, NS) fluoxetine and methylcellulose groups. Repeated measures ANOVA revealed that fluoxetine had no effect on body weight in adolescent rats (F_(1,18)_ = 0.826, NS), but significantly reduced adult body weight (F_(1,18)_ = 9.218, p<0.01). Independent Student t-tests indicated that the body weight reduction in adults was significant (p<0.05) from day 11 of treatment and further on. Similar results were obtained for group 2 and 3 (data not shown).

### Novelty-induced locomotor activity

**Figure 2:**
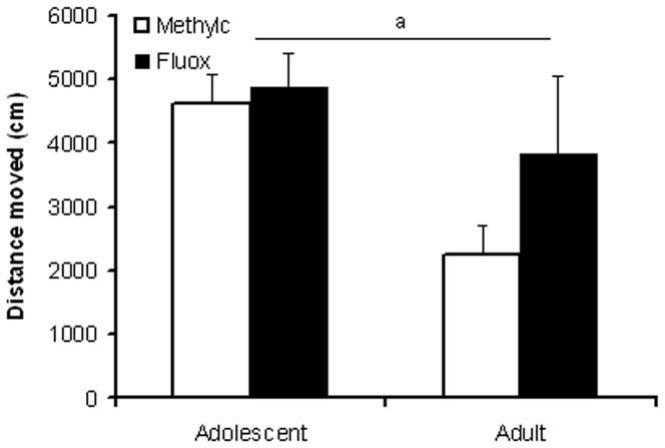
Effect of fluoxetine on exploratory behavior in adolescent and adult rats. Data are presented as mean ± S.E.M. of distance moved within 60 min (n = 10). 7 Days following chronic fluoxetine treatment (12 mg/kg) exploratory behavior in the open field was not affected in adolescent, nor adult, rats. ^a^p<0.05 main age effect.

Two-way ANOVA revealed that total distance moved was significantly higher in adolescent compared to adult rats (F_(3,36)_ = 5.073, p<0.05). However, fluoxetine did not affect locomotor activity (F_(3,36)_ = 0.687, NS) (figure 2), and no age x treatment interaction was observed (F_(3,36)_ = 1.662, NS).

### EPM

**Figure 3:**
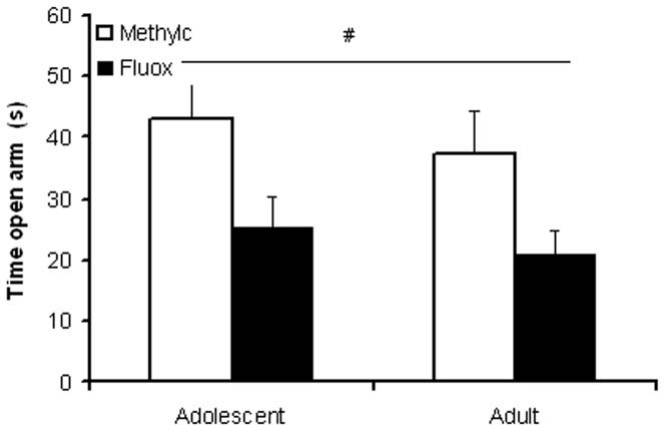
Effect of fluoxetine treatment on anxiety in adolescent and adult rats. Data are presented as mean ± S.E.M. of time spent in the open of the elevated plus maze (n = 10). 10 Days following chronic fluoxetine treatment (12 mg/kg) anxiety in the elevated plus maze test was increased in both adolescent and adult rats. ^#^p<0.05 main treatment effect.

Fluoxetine significantly decreased open arm time in adolescent and adult rats (F_(3,36)_ = 9.344, p<0.005), but there was no significant age (F_(3,36)_ = 0.803, NS) nor age x treatment (F_(3,36)_ = 0.17, NS) effect (figure 3).

### Forced swim test

**Figure 4:**
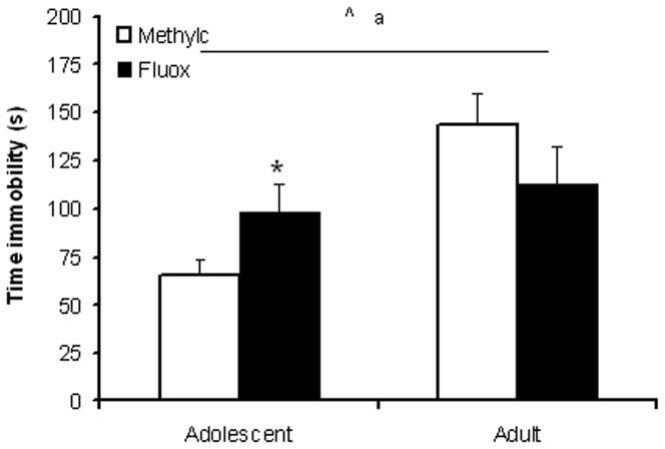
Effect of fluoxetine treatment on behavioral despair in adolescent and adult rats. Data are presented as mean ± S.E.M. of time spent on immobility (n = 10). 10 Days following chronic fluoxetine treatment (12 mg/kg) behavioral despair, expressed as immobility in the forced swim test, was increased in adolescent, but unaffected in adult rats. ^a^p<0.05 main age effect; ^∧^p<0.05 age x treatment interaction; ^*^p<0.05 fluoxetine *versus* methylcellulose in age group.

Adolescent rats spent less time on floating (immobility) compared to adult rats (F_(3,36)_ = 12.544, p<0.001). Further, we obtained a significant age x treatment interaction (F_(3,36)_ = 5.467, p<0.05), but no significant treatment effect was observed (F_(3,36)_ = 0.004, NS) (figure 4). A subsequent Student t-test for time spent on immobility indicated that fluoxetine increased immobility in adolescent rats (t_(1,18)_ = 2.107, p<0.05), but had no effect in adults (t_(1,18)_ = 1.385, NS).

### Startle response and PPI

**Figure 5:**
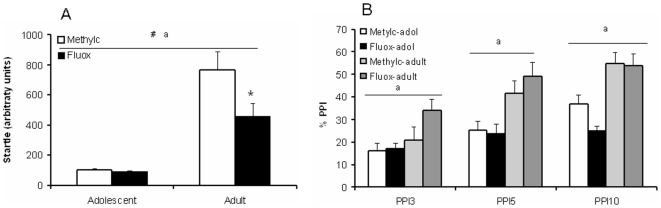
Effect of fluoxetine treatment on the startle reflex and PPI in adolescent and adult rats. Data are presented as mean ± S.E.M. of the startle reflex (n = 10; A) and PPI (n = 9; B). 7 Days following chronic fluoxetine treatment (12 mg/kg) the acoustic startle response was reduced in adult, but not adolescent rats. Fluoxetine had no significant effects on PPI. ^a^p<0.05 main age effect; ^#^p<0.05 main treatment effect; ^*^p<0.05 fluoxetine *versus* methylcellulose in age group.

Overall, the adult animals showed a higher startle reflex compared to the adolescent rats (F_(3,36)_ = 49.006, p<0.0001), there was a significant treatment (F_(3,36)_ = 4.570, p<0.05), and age x treatment (F_(3,36)_ = 4.465, p<0.05) effect (figure 5A). A subsequent Student t-test revealed that fluoxetine decreased the startle reflex in adults (T_(1,18)_ = 2.138, p<0.05) but not in adolescents (T_(1,18)_ = 0.731, NS).

When the animals were exposed to prepulse stimuli of 73 (PP3), 75 (PP5) or 80 (PP10) dB preceding the startling stimulus, four animals had to be removed from the analysis because PPI scores were negative. At PP3, PPI was higher in adult compared to adolescent rats (F_(3,32)_ = 5.755, p<0.05), but no significant treatment (F_(3,32)_ = 2.395, NS) or age x treatment (F_(3,32)_ = 1.956, NS) effect was observed (figure 5B). Similar patterns were found for PP5 [age: (F_(3,34)_ = 14.691, p<0.0001), treatment: (F_(3,34)_ = 0.287, NS), age x treatment: (F_(3,34)_ = 0.711, NS)] and PP10 [age: (F_(3,32)_ = 24.511, p<0.0001), treatment: (F_(3,32)_ = 1.900, NS), age x treatment: (F_(3,32)_ = 1.304, NS)].

### Sleep/wake behavior

**Figure 6:**
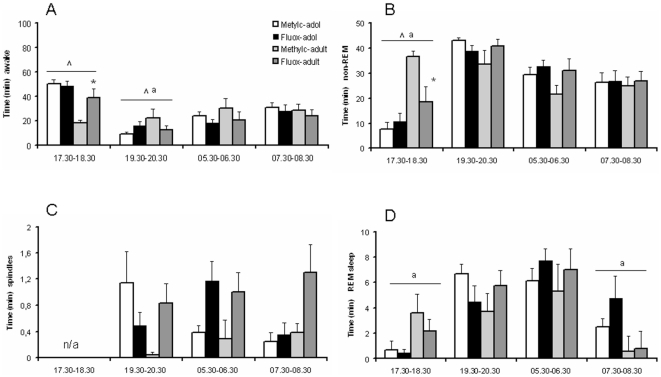
Effect of fluoxetine treatment on the awake state, non-REM sleep, spindles, and REM sleep in adolescent and adult rats. Data are presented as mean ± S.E.M. minutes time spent in the awake state (A), non-REM sleep (B), spindles (C), and REM sleep (D) (n = 4–7). These states were measured at four time intervals: 17.30–18.30 p.m., 19.30–20.30 p.m., 5.30–6.30 a.m. and 7.30–8.30 a.m. Rats were housed under a reversed 12 hr day/light cycle, with lights on at 19.00 p.m. 14–17 Days following chronic fluoxetine treatment (12 mg/kg) wakefulness was increased and non-REM sleep was decreased in adult, but not adolescent rats during the 17.30–18.30 p.m. interval. ^∧^p<0.05 age x treatment interaction; ^a^p<0.05 age effect; ^*^p<0.05 fluoxetine effect significantly different from methylcellulose effect.

3 Animals of the adult methylcellulose treated group were lost due to technical problems. Analysis of the time spent in the awake state across the four consecutive time points (figure 6A) revealed no overall age (F_(1,21)_ = 1.900, NS), treatment (F_(1,21)_ = 0.168, NS) and age x treatment interaction (F_(1,21)_ = 0.337, NS) effects, but the time x age (F_(3,21)_ = 5.920, p<0.001) and time x age x treatment interactions (F_(3,21)_ = 3.146, p<0.05) were significant. Posthoc analysis of the time x age x treatment interaction indicated that there were age x treatment interactions within the 17.30–18.30 (F_(3,21)_ = 15.173, p<0.05) and 19.30–20.30 (F_(3,21)_ = 5.745, p<0.05) intervals. The treatment effects in the 17.30–18.30 interval was significant for the adult (t_(1,9)_ = 2.809, p<0.05), but not adolescent (t_(1,9)_ = 0.346, NS) rats. Thus, fluoxetine increased the awake state in adult rats only. For the 19.30–20.30 interval Student's t-test did not reveal treatment effects for either the adult or adolescent rats (t<1.9). An age effect was obtained for the 17.30–18.30 interval (F_(3,21)_ = 17.613, p<0.05), reflecting increased time spent in the awake state in adolescent rats. Finally, one-way ANOVA (using time as between-subject factor) revealed a significant effect of time (F_(3,96)_ = 20.482, p<0.0001). According to a subsequent Bonferroni post-hoc test time in the awake state was significantly higher in the 17.30–18.30 interval compared to the other intervals (p<0.005), and significantly lower in the 19.30–20.30 interval compared to the 07.30–08.30 interval (p<0.005).

Non-REM sleep analysis revealed an overall age effect (F_(1,21)_ = 4.587, p<0.05), without treatment (F_(1,21)_ = 0.115, NS) and age x treatment effect (F_(1,21)_ = 0.034, NS) (figure 6B). Further, the time x age (F_(3,21)_ = 6.794, p<0.0001) and time x age x treatment interactions (F_(3,21)_ = 3.481, p<0.05) were significant. Two-way ANOVA for each interval separately showed that the time x age x treatment interaction was due to the 17.30–18.30 interval: a significant age x treatment (F_(3,21)_ = 5.455, p<0.05) effect and age effect (F_(3,21)_ = 17.226, p<0.05) was obtained. A Student t-test for the 17.30–18.30 interval showed that fluoxetine decreased non-REM sleep in adult (t_(1,9)_ = 2.732, p<0.05), but not adolescent (t_(1,9)_ = 0.657, NS), rats. The age x treatment interaction for the 19.30–20.30 interval just missed significance (F_(3,21)_ = 4.151, p = 0.054). Finally, there was a significant time effect (F_(3,96)_ = 22.005, p<0.0001), and post-hoc testing indicated that time in non-REM sleep was significantly lower in the 17.30–18.30 interval (p<0.005) and significantly higher in the 19.30–20.30 interval (p<0.005) compared to the 05.30–06.30 and 07.30–08.30 intervals. There was no difference between the later two intervals.

Regarding the time spent in spindles, there were no overall age (F_(1,21)_ = 2.717, NS) and treatment (F_(1,21)_ = 0.553, NS) effects, but the overall age x treatment interaction was significant (F_(1,21)_ = 4.689, p<0.05) (figure 6C). Further, we obtained a significant time x age x treatment interaction (F_(2,21)_ = 3.780, p<0.05). Subsequent analysis revealed a strong trend for an age x treatment interaction during the 19.30–20.30 interval (F_(3,21)_ = 4.264, p = 0.051). No time effect for spindles was observed (F_(2,72)_ = 0.537, NS).

Overall REM sleep analysis did not reveal age (F_(1,21)_ = 0.320, NS), treatment (F_(1,21)_ = 0.604, NS) and age x treatment (F_(1,21)_ = 0.598, NS) effects (figure 6D). There was a significant time x age interaction (F_(3,21)_ = 3.615, p<0.05), and a subsequent two-way ANOVA test for the separate intervals showed age effects for the 17.30–18.30 (F_(3,21)_ = 13.289, p<0.05) and 07.30–08.30 (F_(3,21)_ = 5.232, p<0.05) intervals: REM sleep is reduced in adolescent rats in the 17.30–18.30 interval, and increased in the 07.30–18.30 interval. Finally, a significant effect for time (independent of age or treatment) was obtained (F_(3,96)_ = 11.406, p<0.05), and post-hoc testing revealed that time in REM sleep was significantly lower in the 17.30–18.30 and 07.30–08.30 intervals (p<0.005) compared to the 19.30–20.30 and 05.30–06.30 intervals.

### Fluoxetine and norfluoxetine levels in blood plasma

5 Hrs after the last fluoxetine injection fluoxetine was detected at levels of 260±14 ng/ml in adolescents and 375±38 ng/ml in adults (table 2). Norfluoxetine levels were 1.8 times higher in adolescents (463±55) and 2.9 times higher in adults (1069±85). 1 Week after the last fluoxetine injection fluoxetine levels were below the detection threshold.

**Table 2:** Fluoxetine (ng/ml) and norfluoxetine (ng/ml) levels in blood plasma.

AGE	Fluoxetine5 hr after last injection	Fluoxetine7 d after last injection	Norfluoxetine5 hr after last injection	Norfluoxetine7 d after last injection
**Adult**	375±37	0	1069±85	0
**Adolescent**	260±14	0	463±55	0

### Immunohistochemistry: 5-HT_1A_ receptor and PSA-NCAM in the dorsal raphe nucleus, mPFC, and amygdale

**Figure 7:**
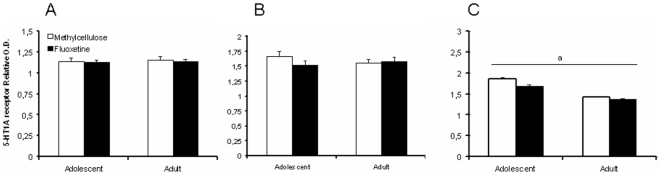
The relative optical density of 5-HT_1A_ receptor immunostaining in the dorsal raphe nucleus, mPFC, and amygdala of fluoxetine and methylcellulose treated adolescent and adult rats. Data are presented as mean ± S.E.M. of O.D. in the dorsal raphe nucleus (A), mPFC (B), and amygdala (C) (n = 4–5) per 100×100 µm. 14–17 Days following chronic fluoxetine (12 mg/kg) treatment no differences in 5-HT_1A_ immunostaining were found in the dorsal raphe nucleus, mPFC and amygdala. Yet, 5-HT_1A_ receptor immunoreactivity was lower in adult compared to adolescent rats in the amygdala. ^a^p<0.05 main age effect.

The relative optical density (O.D.) of 5-HT_1A_ receptor immunostaining in the dorsal raphe nucleus was not affected by age (F_(3,14)_ = 0.035, NS), and fluoxetine treatment (F_(3,14)_ = 0.021, NS; figure 7A). Neither an age x treatment interaction was observed (F_(3,14)_ = 0.085, NS). Likewise, in the mPFC there were no treatment (F_(3,14)_ = 0.576, NS) and age (F_(3,14)_ = 0.043, NS) effects, and there was no significant age x treatment interaction (F_(3,14)_ = 1.276, NS; figure 7B). In contrast, in the amygdala 5-HT_1A_ receptor immunostaining was decreased in adults rats compared to adolescent rats (F_(3,14)_ = 39.566, p<0.0001), but there were no treatment (F_(3,14)_ = 3.239, NS) or age x treatment interaction (F_(3,14)_ = 1.236, NS; figure 7C) effects.

**Figure 8:**
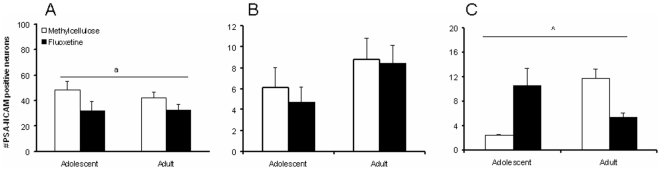
PSA-NCAM immunoreactivity in the dorsal raphe nucleus, mPFC, and amygdala of fluoxetine and methylcellulose treated adolescent and adult rats. Data are presented as mean ± S.E.M. of the numer of immunoreactive neurons in the dorsal raphe nucleus (A), mPFC (B), and amygdala (C) (n = 4–5) per 100×100 µm. 14–17 Days following chronic fluoxetine (12 mg/kg) treatment the number of PSA-NCAM immunoreactivity was lower in adult compared to adolescent rats, but only in the dorsal raphe nucleus. In addition, we obtained a significant age x treatment interaction for PSA-NCAM immunoreactivity in the amygdala, which tended to be increased in adolescent, and decreased in adult rats. ^a^p<0.05 main age effect; ^∧^p<0.05 age x treatment interaction.

PSA-NCAM immunoreactivity in the dorsal raphe nucleus was lower in adult compared to adolescent rats (age: F_(3,14)_ = 7.524, p<0.05), but no treatment effect (F_(3,14)_ = 2.972, NS) or age x treatment interaction (F_(3,14)_ = 0.003, NS) was observed (figure 8A). In the mPFC no significant age (F_(3,14)_ = 2.977, NS), treatment (F_(3,14)_ = 0.245, NS), and age x treatment (F_(3,14)_ = 0.66, NS) effects were found (Figure 8B). Interestingly, in the amygdala we obtained a significant treatment x age interaction (F_(3,14)_ = 6.123, p<0.05; figure 8C) for PSA-NCAM. No further age (F_(3,14)_ = 0.328, NS) and treatment (F_(3,14)_ = 0.227, NS) effects were found. A subsequent Student t-test revealed that fluoxetine tended to increase PSA-NCAM immunoreactivity in adolescent rats (t_(1,8)_ = 1.503, NS) and tended to decreased it in adult rats (t_(1,8)_ = 1.802, NS), but no significant effects were obtained.

## Discussion

Here we show that fluoxetine exerts age-dependent effects: adolescent, as opposed to adult, fluoxetine exposure resulted in an increase in depression-like behavior. In addition, the beneficial effect of fluoxetine on wakefulness was only seen in adult rats. On the other hand, adolescent rats showed resilience to the anorexic effects of fluoxetine [36]; [37]. The age-dependent behavioral effects of fluoxetine are likely to be specific, because novelty-induced locomotor activity was unaffected by fluoxetine in both age groups. We tested the animals 7-14 days after the last fluoxetine injection. Since fluoxetine was undetectable in blood plasma 1 week after the last injection, we argue that these behavioral manifestations reflect neuroplastic changes. As such, PSA-NCAM immunoreactivity in the amygdala at 17 days after the last injection was differentially affected in fluoxetine treated adolescent and adult rats. Given the central role of the amygdala in the modulation of emotional responses [38], the age-dependent effects of fluoxetine on anxiety- and depression-like symptoms may be attributed to changes in amygdalar neuroplasticity.

Our findings correspond to the findings of Oh and colleagues [12], who showed that juvenile mice treated with fluoxetine displayed paradoxical anxiogenic responses. However, these responses disappeared upon drug discontinuation, while the anxiogenic response in our study was observed 1 week after drug discontinuation. Unlike Oh and colleagues [12], we also observed an anxiogenic response in adult rats. Increased anxiety on the EPM test following chronic fluoxetine treatment has also been reported by others [39]; [40]. Iñiguez and colleagues [14] found in Sprague-Dawley rats that anxiety on the EPM was increased at 1 day and 30 days of withdrawal from adolescent fluoxetine exposure, suggesting that the anxiogenic effects of adolescent fluoxetine exposure have a long-lasting nature. Iñiguez et al. [14] further reported that adolescent fluoxetine exposure was associated with antidepressant effects, while we observed depression-like effects in the present study. Rats were treated at PND35–49 in the study of Iñiguez et al. [14] and at PND 25–49 in the current study. It is plausible that the younger age in this study explains this discrepancy, as neonatal (PND4-PND21) fluoxetine exposure also leads to depression-like symptoms during adulthood [41]. Further, given that fluoxetine plasma levels following a 10 mg/kg intraperitoneal injection are approximately twice the blood levels found following oral administration [42], injection route might also explain the discrepancy. Moreover, it is possible that rat strain differences (Sprague-Dawley [14] *versus* WU [present study]) explain the differential outcomes. Finally, Norcross and colleagues [10] reported that chronic adolescent fluoxetine exposure (3–7 weeks of age) in mice did not induce changes in anxiety- and depression-like behavioral responses. Possibly species differences in the pharmacology/metabolism of fluoxetine explain the discrepancy between this study and ours.

While it has been well established that SSRIs decrease REM sleep in both humans and rats [43]–[47], we found no changes in REM sleep in the adolescent and adult rats. This may be explained by the wash-out period in the present study, since the REM sleep suppressive effects diminish after withdrawal from chronic SSRI treatment within a few days, and in some of the discontinuation nights a REM rebound was found [48]; [49]. In addition, it is possible that the Wistar strain we used (WU) is relatively insensitive to the REM sleep-reducing effects of fluoxetine, given that there are rat strain differences regarding sleep regulation [50]. Nonetheless, the sleep/wake pattern was clearly affected by fluoxetine in adult rats, while it had no effects in adolescent rats. The increased waking and decreased non-REM sleep in the period before the lights were switched on (17.30–18.30 p.m.) suggests that wakefulness was increased, as has been shown previously [49]. The differences occurred in the last hr of the dark period, when the amount of non-REM sleep is rather low. During the period in which deep non-REM sleep is prevalent, the light period and especially the early hrs of the light period, there were no differences between the age groups. The increased amount of waking in adolescent rats agrees with the commonly reported age-related changes in sleep quantity [51].

There was a significant decrease in basal startle response in the fluoxetine-treated adult rats compared to the adult controls, an effect that was not found in the adolescent rats. Shanahan and colleagues [52] also reported a reduction in the startle response upon chronic fluoxetine treatment in adult mice. Apparently, this effect does not extend to adolescence, which indicates that the pathway underlying the acoustic startle response and the stress responses measured in the elevated plus maze and forced swim tests differ. Yet, it should be noted that there could have been a floor effect in adolescent rats, because their startle response under control conditions was already low. Further, PPI was not significantly reduced in adolescent and adult rats, which is in line with previous observations in adult mice [52]. PPI is modulated by a variety of 5-HT receptors, including the 5-HT_1A_, 5-HT_1B_, and 5-HT_2A_ receptors [52]–[57]. We did not observe changes in 5-HT_1A_ receptor immunoreactivity in the amygdala. Although PPI is mediated by several other brain areas, it could be one reason why fluoxetine failed to significantly affect PPI.

5 Hrs after the last fluoxetine injection, the fluoxetine concentration in blood plasma was 260±14 ng/ml in adolescents and 375±38 in adults, which falls in the range of a low 50–60 to a high 400–500 ng/ml reported in humans [58]–[61]. Body weight of the fluoxetine adolescent rats was 220 grams and fluoxetine adult rats 360 grams at the last treatment day, i.e. 5 hrs before blood collection. Correction for body weight therefore reveals nearly identical fluoxetine concentrations (adolescent 1.18 *versus* adult 1.04 ng/ml/gram body weight fluoxetine). In line with this, it has been shown that pediatric brain levels of fluoxetine are not significantly different from typical adult levels when corrected for the effects of dose per mass [62]. Further, the norfluoxetine/fluoxetine ratio in adolescents was 1.8, which approaches the 1.3–1.5 ratio measured in humans [60]. The higher ratio of 2.9 in adults may relate to a lower metabolism of norfluoxetine. Because both fluoxetine and norfluoxetine (the most active metabolite of fluoxetine [63] levels were below the detection threshold at the time of behavioral testing we do not expect that the differential norfluoxetine/fluoxetine ratio's in adult and adolescent rats affected the behavioral outcomes. It is therefore most likely that the age-dependent effects of fluoxetine on behavior are due to neuroplastic changes.

As reported previously [25], fluoxetine treatment in adult rats tended to reduce PSA-NCAM immunoreactivity in the amygdala. Interestingly, we show for the first time that fluoxetine tended to increase PSA-NCAM immunoreactivity in adolescent rats. Although these effects were not significant, the age x treatment interaction was. Incorporation of PSA confers anti-adhesive properties to NCAM [64], which allows neurons to participate in plastic events such as neurite outgrowth or synaptic reorganization [65]. Varea and colleagues [25] showed that decreases in PSA-NCAM following fluoxetine treatment were not correlated with alterations in synaptophysin immunoreactivity in the basolateral amygdala and mPFC. Synaptophysin is a marker for presynaptic boutons, suggesting that the age-dependent neuroplastic effects as we observed in the amygdala do not involve changes in neurotransmitter release. As antidepressant treatment can prevent amygdalar dendritic hypertrophy induced by chronic stress [66], increased synaptic remodelling in adolescent fluoxetine exposed rats may resemble effects of chronic stress. Yet, this is highly speculative at this point and the exact implications of our findings thus remain to be investigated. Given that PSA-NCAM is preferentially involved in neurite and spine outgrowth [67], it would be of interest to study the morphological changes associated with the age-dependent effects of fluoxetine. In contrast to previous studies [25], [26] we did not observe effects of chronic fluoxetine treatment on PSA-NCAM immunoreactivity in the mPFC. A potential explanation is the fact that our measurements were conducted 2.5 weeks following the last fluoxetine injection, while increases in fluoxetine-induced PSA-NCAM expression were observed 1 day following chronic fluoxetine treatment [25], [26]. In the dorsal raphe nucleus we observed an age-dependent effect for PSA-NCAM, in line with the idea that aging is associated with a decrease in synaptic remodelling [68], although the adult rats in the present study were still quite young. 5-HT_1A_ receptor immunostaining in the dorsal raphe nucleus, amygdala and mPFC was not differentially affected by fluoxetine treatment. This finding was somewhat surprising, given the role of the 5-HT_1A_ receptor in the antidepressant effects of SSRIs [27]; [28] and the interaction between 5-HT_1A_ receptor activation and PSA-NCAM immunoreactivity [29]. Yet, it does not exclude the involvement of the 5-HT_1A_ receptor, because we did not assess 5-HT_1A_ receptor function. We did observe that 5-HT_1A_ immunostaining was lower in adult rats compared to adolescents in the amygdala, which may be in line with the region-independent decline in 5-HT_1A_ receptor immunoreactivity in mice and human during ageing [69]. But again, our adult rats were relatively young. Finally, it has been previously observed that the 5-HT_3_ receptor colocalizes with PSA-NCAM in the prefrontal cortex, and that the 5-HT_3_ receptor antagonist ondansetron reversed the effects of chronic fluoxetine treatment on PSA-NCAM expression in the mPFC [26], suggesting that the 5-HT_3_ receptor is an important target for future research.

A possible limitation of the present study is that we used commercial animals, both the adolescent and adult rats were shipped 4 days before the start of the fluoxetine treatment. It is conceivable that the adolescent rats were more shipping stress sensitive than adult rats. Yet, given that the adolescent were more active in the Phenotyper, were less immobile in the forced swim test and showed a reduced acoustic startle response compared to adult rats it is not a likely confounding factor. Nonetheless, having more time for acclimatisation following shipping would have been more ideal. Further, because we conducted two behavioral tests in the same group of animals (see table 1) it is possible that stress associated with the first test affected performance in the second test. However, no relationship was found for fluoxetine effects in adolescent and adults rats in the open field and elevated plus maze tests. This was also true for the acoustic startle/PPI and forced swim test data. Likewise, the absence of an anorexic effect in adolescent rats was not indicative for reduced efficacy of fluoxetine in these animals. Another issue is that immunohistochemistry was executed on brains derived from animals that were orally treated and tested in the acoustic startle/PPI and forced swim test. Although stress induces neuroplastic changes, it is critical to note that all animals received the same amount of stress. Notable is also that we used healthy male rats for the present study. Although our observations may correspond to reports in humans that fluoxetine treatment in adolescents may paradoxically increase depression-related behavior without notification of gender differences [4]; [7], future research is needed to elucidate the age-dependent effects of SSRIs in depression-related animal models as a function of sex. Finally, due to some technical problems during cutting our sample size for the immunohistochemical studies was rather small. Yet, we obtained a significant age x treatment interaction effects and it was clear that fluoxetine differentially affected PSA-NCAM immunoreactivity in adolescent and adult rats. It is also important to note that the brains that had been cut correctly were not pre-selected brains based on behavioral performance.

In conclusion, we show that adolescent fluoxetine exposure can lead to an adverse increased depression-like outcome. In addition, adolescents may not benefit from fluoxetine's effects on wakefulness. *Vice versa*, the anorexic effects of fluoxetine were not seen in adolescent rats. These age-dependent effects of fluoxetine on emotional behavior are most likely due to neuroplastic changes, since amygdalar PSA-NCAM was decreased in adults, but increased in adolescent rats. Together, the data importantly contribute to the debate about the safety of SSRIs in adolescents and the experiment set-up as used here may help in the identification of the mechanisms underlying the age-dependent effects of fluoxetine.
